# Identification of the novel bacterial blight resistance gene *Xa46*(t) by mapping and expression analysis of the rice mutant H120

**DOI:** 10.1038/s41598-020-69639-y

**Published:** 2020-07-28

**Authors:** Shen Chen, Congying Wang, Jianyuan Yang, Bing Chen, Wenjuan Wang, Jing Su, Aiqing Feng, Liexian Zeng, Xiaoyuan Zhu

**Affiliations:** Guangdong Provincial Key Laboratory of High Technology for Plant Protection, The Plant Protection Research Institute of Guangdong Academy of Agricultural Sciences, Guangzhou, 510640 China

**Keywords:** Agricultural genetics, Plant breeding

## Abstract

Rice bacterial leaf blight is caused by *Xanthomonas oryzae* pv. *oryzae* (*Xoo*) and produces substantial losses in rice yields. Resistance breeding is an effective method for controlling bacterial leaf blight disease. The mutant line H120 derived from the *japonica* line Lijiangxintuanheigu is resistant to all Chinese *Xoo* races. To identify and map the *Xoo* resistance gene(s) of H120, we examined the association between phenotypic and genotypic variations in two F_2_ populations derived from crosses between H120/CO39 and H120/IR24. The segregation ratios of F_2_ progeny consisted with the action of a single dominant resistance gene, which we named *Xa46*(t). *Xa46*(t) was mapped between the markers RM26981 and RM26984 within an approximately 65.34-kb region on chromosome 11. The 12 genes predicted within the target region included two candidate genes encoding the serine/threonine-protein kinase Doa (Loc_Os11g37540) and Calmodulin-2/3/5 (Loc_Os11g37550). Differential expression of H120 was analyzed by RNA-seq. Four genes in the *Xa46*(t) target region were differentially expressed after inoculation with *Xoo*. Mapping and expression data suggest that Loc_Os11g37540 allele is most likely to be *Xa46*(t). The sequence comparison of *Xa23* allele between H120 and CBB23 indicated that the *Xa46*(t) gene is not identical to *Xa23*.

## Introduction

Rice (*Oryza sativa*) bacterial blight which caused by the pathogen *Xanthomonas oryzae* pv. *oryzae* (*Xoo*) is one of most serious three rice disease in the world, and limits rice productivity each year owing to its high epidemic potential and the lack of effective bactericides^1,2^. *Xoo* causes a systemic infection of the vascular system that results in yellowish brown long strip or offwhite lesions along leaf veins at the maximum booting stage. Rice infected by *Xoo* can lose 10–20% and even up to 80% of its yield^3,4^. Rice bacterial blight disease is usually prevalent in tropical subtropical regions rice-growing regions except North America^5,6^.

Normally plant disease resistance is divided into qualitative (complete) or quantitative (partial) according to the plant’s specific interactions against pathogen invasion^7^. Qualitative resistance belongs to pathogen race-specific resistance which controlled by major resistance (MR) genes. Quantitative resistance belongs to pathogen race-nonspecific resistance which is generally mediated by multiple minor genes or quantitative trait loci (QTLs)^8^. The rice-*Xoo* pathosystem as a host–pathogen interactions and co-evolution genetic model was used to dissect plant disease resistance mechanisms^5,9^. In the rice-*Xoo* pathosystem, MR-mediated race-specific resistance usually follows the gene-for-gene relationship^9,10^.

MR has been widely applied to rice breeding in consideration of its high level of resistance and easy genetic manipulation. Application of resistance variety is firmly believed to be the most effective and environment-friendly measure to prevent and control bacterial blight disease^1,2^. To date, at least 45 race-specific bacterial blight resistance (R) genes to different *Xoo* races derived from cultivated and wild rice and artificial mutants were identified or mapped^11,12^. However, resistance provided by R genes could break down due to the emergence of new *Xoo* races and rapid changes in the pathogenicity of *Xoo*^1,3,10^. To solve the problem of *Xoo* resistance breakdown, new broad-spectrum resistance genes need to be identified.

RNA-seq is used as a standard method for analyzing gene expression profile, including bacterial infections^13^, such as transcript profiles of the RNA chaperone Hfq in *Salmonella enterica*^14^, *Burkholderia cenocepacia* and *Helicobacter pylori*^15,16^ etc. RNA-seq has explicated the difference of RNA level in lots of diverse plants and bacteria caused by diseases^17,18^. To better understand host plant responses during simultaneous heat and pathogen stress, The experiment that a transcriptomics profile of the *Xoo* resistance gene *Xa7* against *Xanthomonas oryzae* was conducted during high-temperature stress characterized the plant responses genes against coinstantaneous heat and pathogen stress^19^. RNA-seq has proved to be obtainable transitorily, yet this method could have already effectively altered our vision of the breadth and depth via eukaryotic transcriptomes assay, which could improve the efficiency of gene identification as well^20^.

Study on the molecular genetics of mutant lines is an effective approach for new gene discovery and dissection of the biology function mechanism of the plants. In previous research, we identified a new mutant H120 resistant to most of Chinese *Xoo* races. Purpose of this study was to identify resistance gene in the mutant H120 using the methods of genetic mapping and RNA-seq.

## Results

### Resistance reaction to Chinese *Xoo* pathotypes

**Figure 1:**
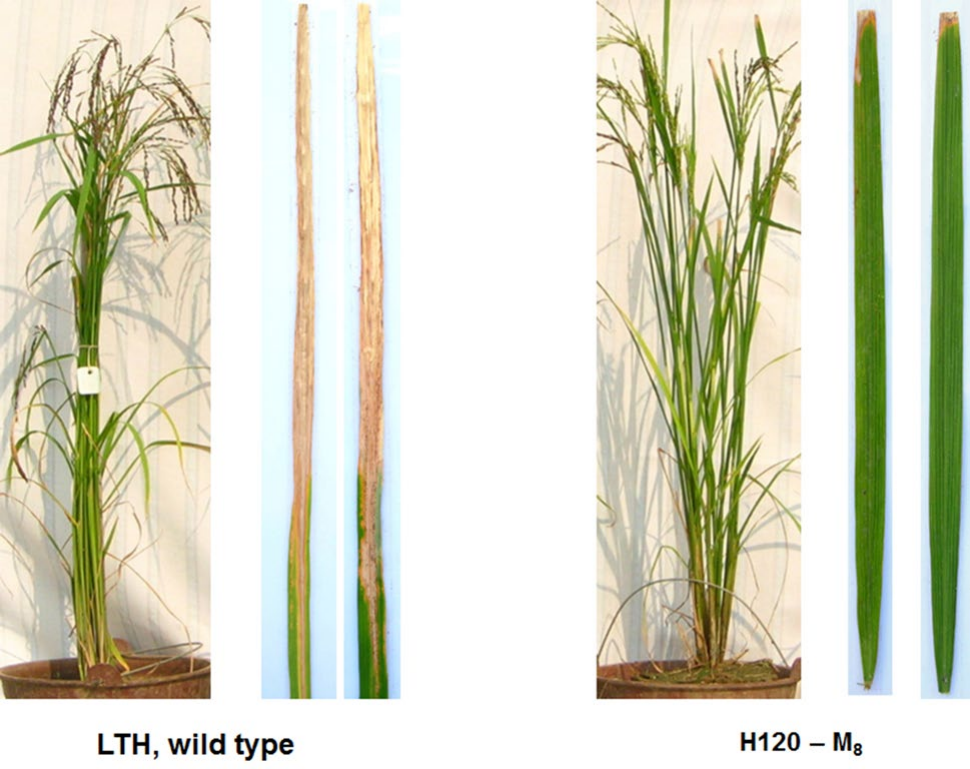
Phenotypes of LTH and H120 against *Xanthomonas oryzae* pv. *oryzae*. Wild type LTH shows highly susceptible to *Xanthomonas oryzae* pv. *oryzae* and the mutant H120 shows highly bacterial blight resistance.

**Figure 2:**
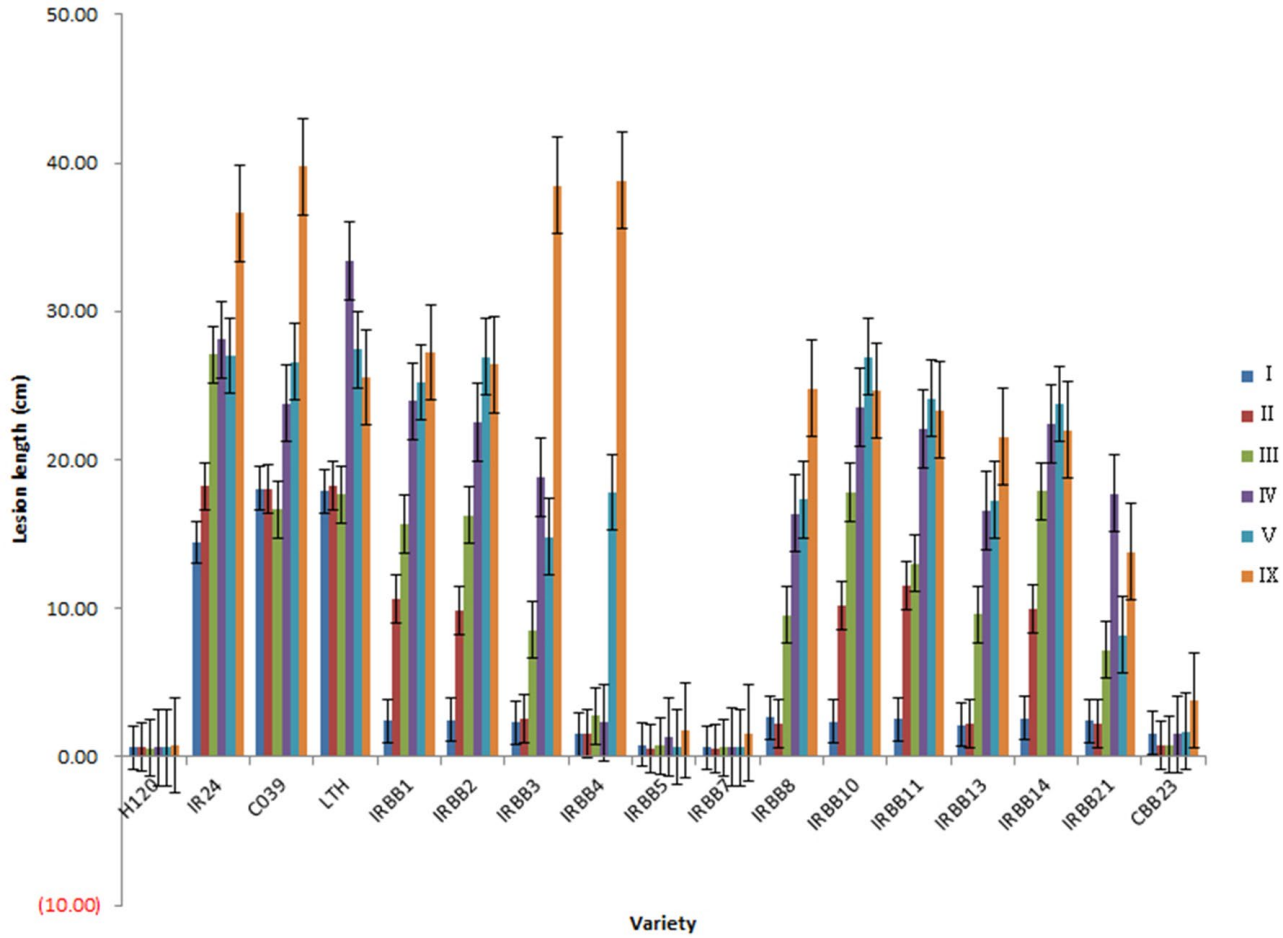
Lesion lengths of 17 varieties after inoculation with six *Xanthomonas oryzae* pv. *oryzae* pathotype isolates (I–V and IX). For each variety, 20 leaves from four individuals were counted to determine the average and error bars represent the standard error.

The mutant line H120 which derived from Lijiangxintuanheigu (LTH) (Fig. 1) mutants and the series of varieties including IR24, CO39, LTH, IRBB1, IRBB2, IRBB3, IRBB4, IRBB5, IRBB7, IRBB8, IRBB10, IRBB11, IRBB13, IRBB14, IRBB21 and CBB23 were used for resistance evaluation against six *Xoo* strains with diverse virulence in South China (Table 1). Among the plants, H120 showed high resistance to all six *Xoo* pathotypes including pathotype I isolate GD9240, pathotype II GD9269, pathotype III GD9279, pathotype IV GD9315, pathotype V GD9352, and pathotype IX GD9385 from South China. The varieties IR24, CO39, and LTH were all susceptible to all six races. IRBB1, IRBB2, IRBB10, IRBB10 and IRBB14 were resistant to race I and susceptible to other races; IRBB3, IRBB8, IRBB13 and IRBB21 were resistant to race I, II and susceptible to other races; IRBB4 was resistant to race I, II, III, IV and susceptible to other races; IRBB5, IRBB7 and CBB23 were resistant to all the races in this study (Table 1; Fig. 2).

**Table 1:** Phenotypes of 17 varieties inoculated with six pathotypes of *Xanthomonas oryzae* pv. *oryzae*.

Varieties	Resistance genes	I	II	III	IV	V	IX
H120	*Xa46*(t)	HR	HR	HR	HR	HR	HR
IR24	–	S	S	HS	HS	HS	HS
C039	–	S	S	S	HS	HS	HS
LTH	–	S	S	S	HS	HS	HS
IRBB1	*Xa1*	R	MS	S	HS	HS	HS
IRBB2	*Xa2*	R	MS	S	HS	HS	HS
IRBB3	*Xa3*	R	R	MS	S	S	HS
IRBB4	*Xa4*	R	R	R	R	S	HS
IRBB5	*xa5*	HR	HR	HR	R	HR	R
IRBB7	*Xa7*	HR	HR	HR	HR	HR	R
IRBB8	*xa8*	R	R	MS	S	S	HS
IRBB10	*Xa10*	R	MS	S	HS	HS	HS
IRBB11	*Xa11*	R	MS	S	HS	HS	HS
IRBB13	*xa13*	R	R	MS	S	S	HS
IRBB14	*Xa14*	R	MS	S	HS	HS	HS
IRBB21	*Xa21*	R	R	MS	S	MS	S
CBB23	*Xa23*	R	HR	HR	R	R	MR

*HR* highly resistant, *R* resistant, *MR* moderately resistant, *MS* moderately susceptible, *S* susceptible, *HS* highly susceptible.

### Resistance inheritance of H120

To analyze the resistance inheritance of the mutant line H120, two genetic populations were constructed by crosses among susceptible varieties IR24, CO39 and the resistant parent H120. We used the *Xoo* predominant race IV isolate GD9315 in South China to inoculate the cross parents and F_1_ progenies at tillering stage. 20 days after inoculation (DAI), the average lesion lengths of the susceptible parents IR24 and CO39 were 28.2 ± 1.1 cm and 22.2 ± 1.2 cm, respectively, while the average lesion length of H120 was 3.5 ± 0.3 at 20 DAI. F_1_ plants derived from the IR24/H120 and CO39/H120 crosses all showed resistance to GD9315, whose average lesion length was 3.7 ± 0.3 cm. Mapping populations from the IR24/H120 and CO39/H120 crosses for genetic analysis were used to dissect resistance genetic of H120. We used isolate GD9315 to inoculate 1,263 and 3,128 F_2_ individuals from the IR24/H120 and CO39/H120 crosses in the field. According to the Standard Evaluation System for Rice, the segregation ratios of resistant and susceptible F_2_ individuals from the crosses IR24/H120 and CO39/H120 fitted to 3:1 (*X*^2^ = 0.0953, *P* > 0.05 and *X*^2^ = 0.3342, *P* > 0.05, Table 2) with 952 resistant to 311 susceptible and 2,360 resistant to 768 susceptible, respectively, which suggested that H120 harbour a single dominant resistance locus with which temporarily designated *Xa46*(t).

**Table 2:** Resistance response of F_1_ and F_2_ crosses between H120 and IR24(CO39) against isolate GD9315.

Cross	Resistance response of parents		F_1_ resistance response	F_2_ resistance response					
	P1	P2		Resistant	Susceptible	Total	Seg	*X*^*2*^	*P*
IR24/H120	S	R	R	952	311	1,263	3:1	0.0953	0.05
CO39/H120	S	R	R	2,360	768	3,128	3:1	0.3342	0.05

*Seg*. means segregation ratio.

### Molecular identification of the *Xa46*(t) gene

We used bulked segregant analysis (BSA) and recessive class analysis (RCA) to identify the target gene. The resistant pool (RP) and susceptible pool (SP) with equal DNA of 15 resistant and susceptible F_2_ individuals were derived from the IR24/H120 cross, respectively. We first used 350 rice simple-sequence repeat (SSR) markers to screen DNA polymorphisms of two parents and the RP and SP. Two SSR markers of chromosome 11, RM26777 and RM206, displayed clear bulk-specific polymorphisms among the resistant and susceptible parents and the RP and SP. These markers were initially used for linkage analysis in the RP and SP individuals, then tested all the F_2_ individuals. The confirmed results suggested that the *Xa46*(t) gene was mapped to an 8.0 cM region between RM26777 and RM206.

### Preliminarily genetic mapping of *Xa46*(t)

**Figure 3:**
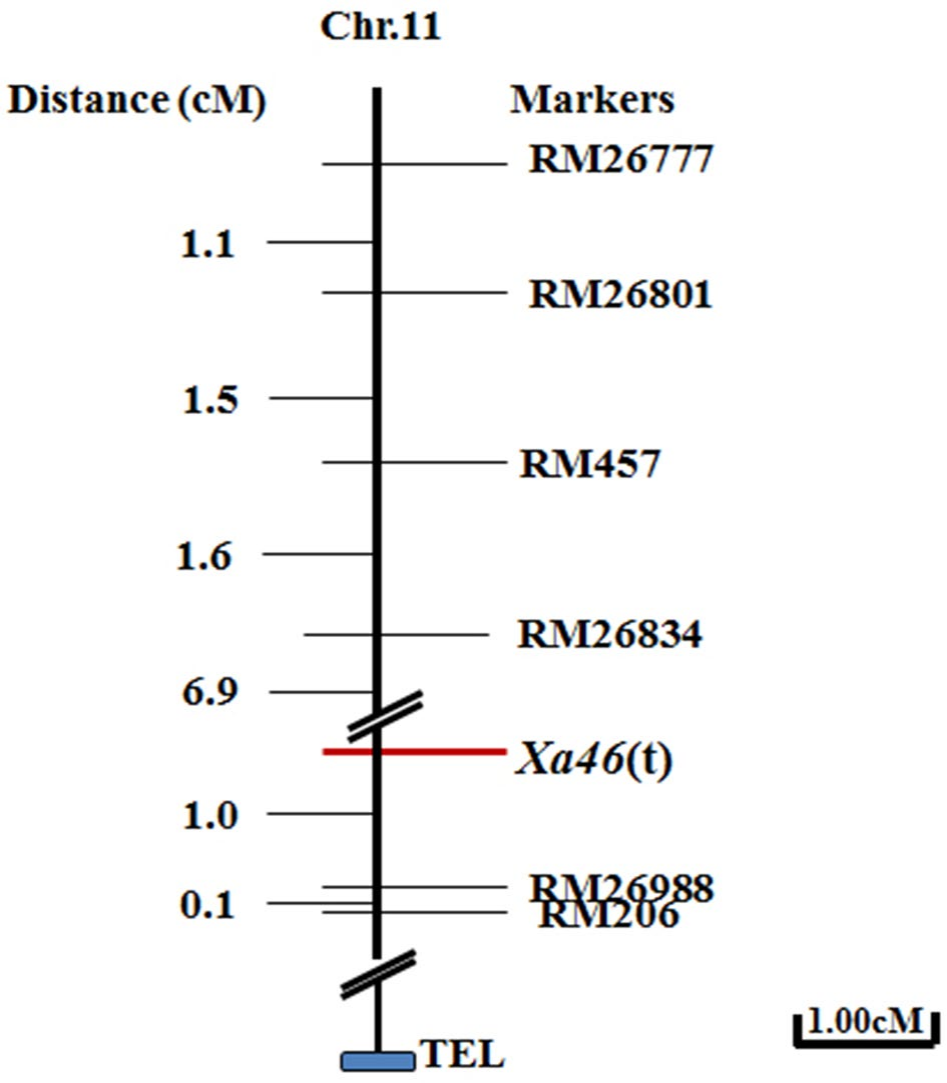
Genetic map of *Xa46*(t) constructed by using 311 F_2_ susceptible individuals. *Xa46*(t) was flanked by simple sequence repeat (SSR) markers RM26834 and RM26988 on chromosome 11. SSR markers (right) were ordered based on recombination data. Genetic distances are indicated in centiMorgans (left).

To map the *Xa46*(t) gene, we selected a set of 52 SSR markers between markers RM26777 and RM206 to further analyze the linkage with the target gene. Of the 52 SSR markers selected, four SSR markers, RM26801, RM457, RM26834 and RM26988, showed polymorphisms between the IR24 and H120 parents. These four markers were used to analyze the linkage distances to *Xa46*(t). The confirmed results showed that three polymorphic markers RM26801, RM457, and RM26834 of the RM26777 side were mapped closer to the target gene with 10.0, 8.5 and 6.9 cM. Another marker RM26988 of the RM206 side was mapped more closer to the target gene with 1.0 cM (Fig. 3). Finally the *Xa46*(t) gene was located in a 7.9 cM zone between RM26834 and RM26988, in which 42 and 6 recombinants were detected from RM26777 and RM206 sites.

### Physical mapping of the *Xa46*(t) gene

**Figure 4:**
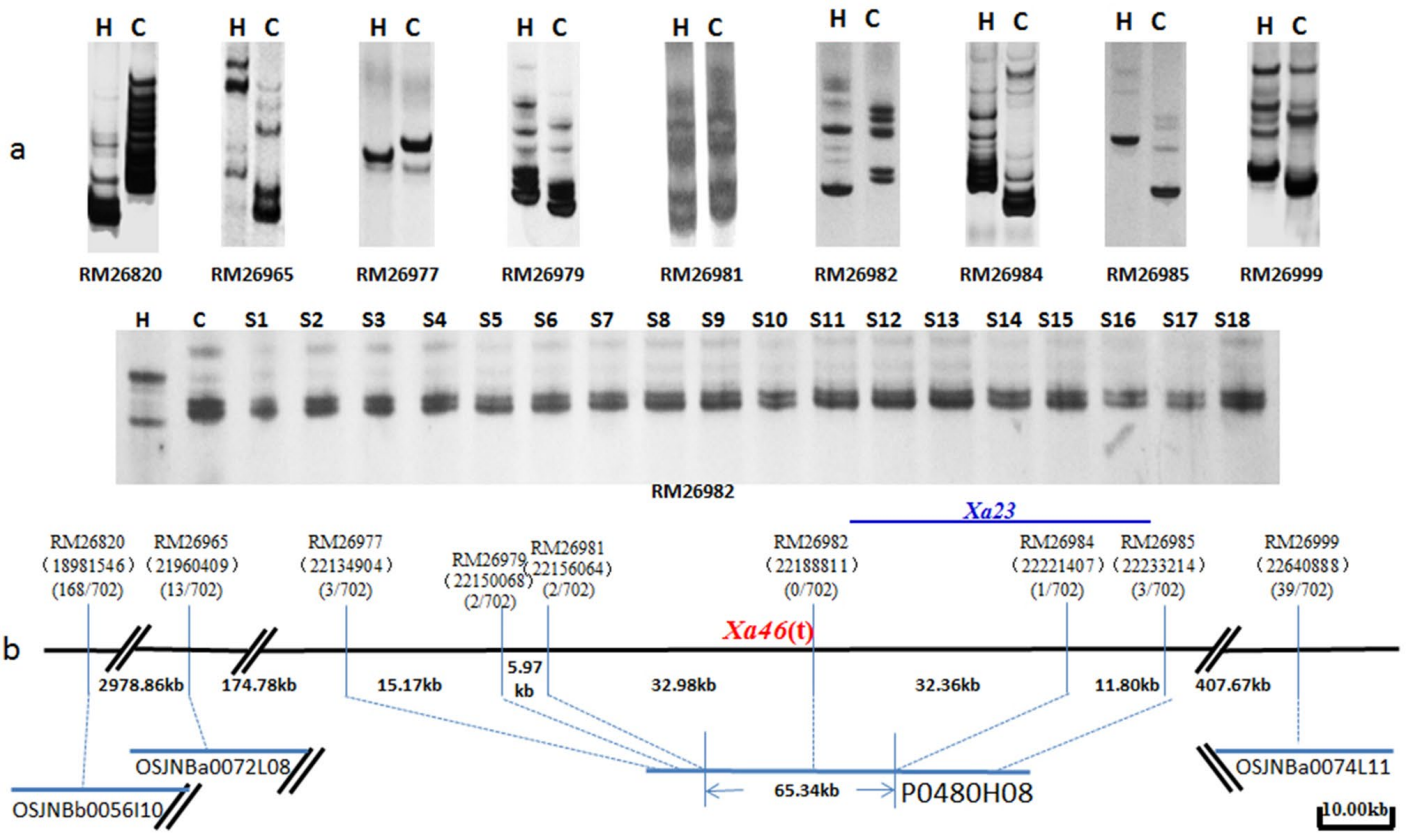
Fine mapping of *Xa46*(t). ^a^Polymorphisms between H120 (H) and C039 (C) were revealed by SSR markers RM26820, RM26965, RM26977, RM26979, RM26981, RM26982, RM26984, RM26985 and RM26999. Molecular genotypes of some susceptible F_2_ plants revealed by RM26982 are also shown. ^b^Physical map of the *Xa46*(t) locus. *Xa46*(t) was mapped between the markers RM26981 and RM26984 on chromosome 11 (11S). The nine markers identified in this study, including the co-segregating maker RM26982. *Xa46*(t) was located in a region corresponding to a 65.34 kb interval in the PAC clone P0480H08 of Nipponbare. *Xa23* was also mapped on this region marked with blue (Wang et al. ^21^. All the linked markers were anchored at the BAC/PAC clones.

To fine map the *Xa46*(t) gene, we selected second round of 60 molecular markers to identify linkages to the target gene in the other F_2_ population from the CO39/H120. A parental polymorphic survey of CO39/H120 was detected first. The test result showed that nine SSR markers with clear polymorphisms were selected as further fine mapping markers within the target region. A total of 702 F_2_ individuals with highly susceptible reaction (lesion length > 10 cm) to the GD9315 isolate were selected to narrow down the objective region of *Xa46*(t). Thirty-two BAC/PAC clones from the rice reference genome sequence of Nipponbare were overlapped *Xa46*(t) within about 3.66 Mb interval (Fig. 4). The anchor markers RM26977, RM26979, RM26981, RM26982, RM26984, and RM26985 were landed on the target PAC clone (P0480H08) at about 98.28 kb physical zone. Recombinants analysis of the 702 F_2_ individuals showed two and one distinct recombinants were identified with RM26981 and RM26984, in which the target gene was fine mapped to a 65.34-kb on clone P0480H08 between RM26981 and RM26984 (Fig. 4). Within the region of interest. The marker RM26982, flanked by RM26981 and RM26984, co-segregated with *Xa46*(t). Coincidentally the *Xa23*(t) gene was also mapped on this region^21^.

### Candidate gene annotation of the *Xa46*(t) gene region

We analyzed the code protein of the *Xa46*(t) gene mapping rengion based on the release 7.0 of the MSU Rice Genome Annotation Project Database and Resource, which that the target zone contains twelve genes with complete structure (Supplementary Table S1). The genes predicted in the region encode several proteins, including ETO1-like protein 1, the serine/threonine-protein kinase Doa, Calmodulin-2/3/5, ADP-ribosylation factor-like protein 5, an uncharacterized expressed protein, two transposon proteins, and five hypothetical proteins. Of these, we considered the two genes (Loc_Os11g37540 and Loc_Os11g37550) encoding the serine/threonine-protein kinase Doa and Calmodulin-2/3/5 to be promising candidate genes conferring resistance to *Xoo* isolate GD9315, as that this two coding genes have been linked with resistance response reported^22^.

### Transcriptome analysis of H120 against bacterial blight

We used default parameters on the HISAT software to analyze the filtered sequences for genomic location analysis. The sequencing data quality evaluation showed that RNA-seq was accurate (Supplementary Table S2). The comparison of RNA-seq reads and reference genomes is shown in Supplementary Table S3. Statistics on the density of total mapped reads to each chromosome on the genome (positive and negative chain), explain the relationship between the number of reads on the chromosome and the length of the chromosome. The results showed that the sequence of RNA-seq reads was well-distributed in the rice genome. In RNA-seq analysis, we estimated gene expression level by the count of the sequencing reads of the genomic region or exon of a given gene. The number of genes at different expression levels and the expression level of individual genes were counted (Supplementary Table S4). The FPKM distribution map and violin map were used to compare the gene expression levels under different experimental conditions. For repeated samples under the same experimental conditions, the final FPKM is the average of all repeated data.

### Differential expression analysis of candidate genes in *Xa46*(t) mapping region

**Figure 5:**
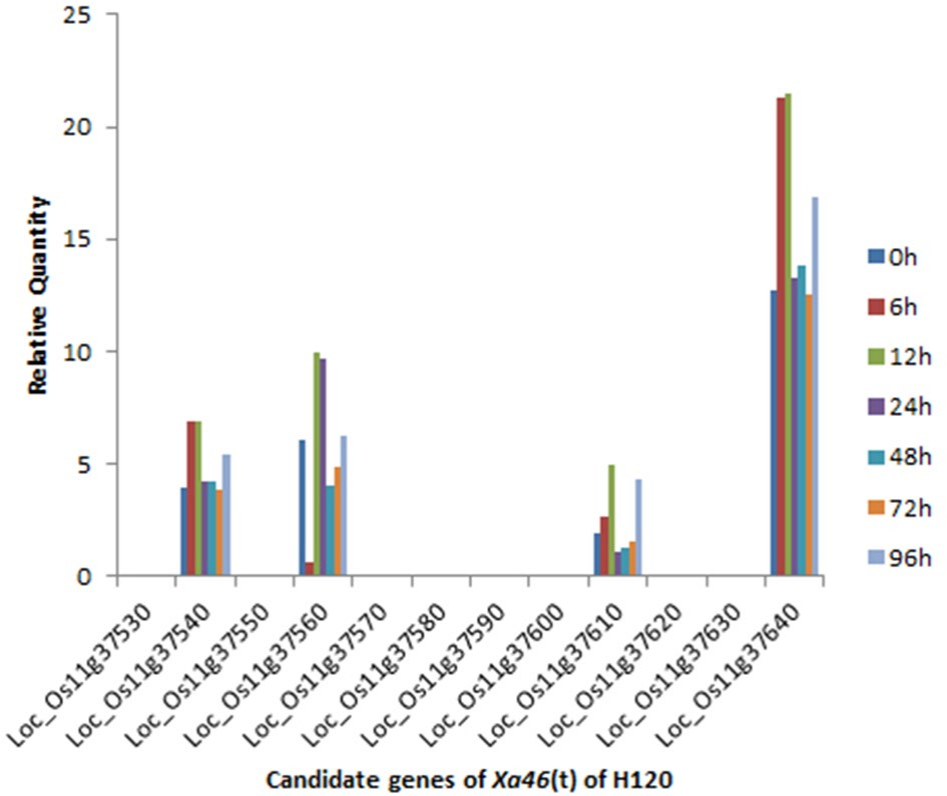
Expression of the *Xa46*(t) candidate genes in the target region were analyzed at seven time-points (0, 6, 12, 24, 48, 72, and 96 h) after inoculation of H120 with the *Xanthomonas oryzae* pv. *oryzae* pathogen.

The input data for differential gene expression analysis were readcount data from gene expression-level analysis. Within the gene mapping region of the bacterial blight gene *Xa46*(t), we analyzed the differential expression of genes from the H120 to confirm which candidate is the possible target of *Xa46*(t). The candidate genes Loc_Os11g37540, Loc_Os11g37560, Loc_Os11g37610 and Loc_Os11g37640 were differentially expressed after inoculation with the pathogen (Fig. 5). Other candidate genes either were not expressed or were expressed at a low level only at a certain timepoint. Combined with the genes’ structure analysis of these four expressed candidate genes, Loc_Os11g37540, the serine/threonine protein kinase Doa, is most likely to be the causal gene for the bacterial blight resistance.

### Sequence analysis of *Xa23* alleles of H120

**Figure 6:**
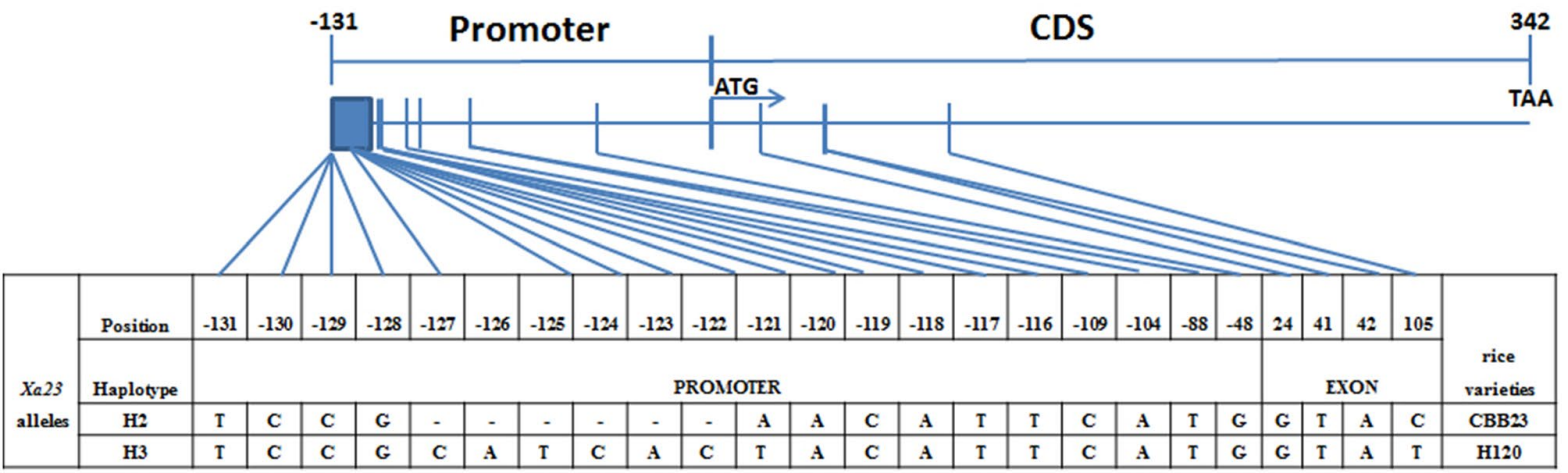
Haplotype analysis of the *Xa23* alleles region between H120 and CBB23. The key domain of *Xa23*/*xa23* alleles contains the promoter region − 131 bp upstream sequences from ATG start codon and the 342-bp coding region shown in graphic on the top. The numbers on the top row shows the positions of nucleotide polymorphisms from the *EBE*/*ebe* to the stop codon TAA. The “–” indicate deletions.

To discriminate the relationship of *Xa46*(t) and *Xa23*, we sequenced the *Xa23* allele of H120, since *Xa46*(t) was mapped on the *Xa23*-linked region. First, we amplified the *Xa23* allele with several pairs of overlapping gene-specific primers and visualized on agarose gels, the result showed that H120 and CBB23 could amplify the target fragments. Then we sequenced and aligned the PCR products of H120 and analyzed alleles nucleotide diversity polymorphism between H120 and CBB23. We detected 28SNP and 23 InDels through comparative analysis with CLUSTAL W in the total *Xa23* gene region (Supplementary Fig. S1). Specially, 3 SNPs and 1 InDel were detected from the *Xa23 EBE*/*ebe* domain to the stop codon TAA about 473 bp (Fig. 6). A 6-bp InDel and 1 SNP polymorphism exist in the promoter *EBE*/*ebe* region of *Xa23* allele between H120 and CBB23, and 1 SNP polymorphism in the *CDS*_*Xa23*_ region. Interestingly, the haplotype of *Xa23* allele in H120 is almost the same to haplotype H3 of *Xa23* allele, in which H3 was defined as susceptible to *Xoo*^23^. So the *Xa46*(t) gene is different from *Xa23*.

## Discussion

In this study we identified a novel rice *Xoo R* gene from the mutant line H120. The target gene was mapped to chromosome 11 based on the linkage analysis. Currently about 45 rice bacterial blight resistance genes have been identified or mapped^11,12,24,25^. Fifteen genes including *Xa3/26* , *Xa4, Xa10*, *Xa21*, *Xa22*, *Xa23*, *xa26*, *Xa30*, *Xa32*, *Xa35*, *Xa36*, *Xa39*, *Xa40*, *Xa43*, *xa44* and *xa44* have been reported to be located on chromosome 11^21,24–30^. The target novel gene name *Xa46*(t) derived from H120, based on the gene nomenclature system of rice *Xoo* R genes^31^, located near the centromeric region of chromosome 11.

Of the 45 identified rice bacterial blight resistance genes^25^, only 11 have been isolated and characterized^30,32,33^. Four bacterial blight resistance genes, *xa5*, *xa13*, *xa25* and *xa41*, are recessive. They possess almost different resistance protein, in which *xa5* encodes the small subunit of transcription factor IIA (TFIIAã)^34^, *xa13* and *xa25* belong to the MtN3/saliva gene family^35^ and *xa41* represents a new resistance allele owing to an 18-bp deletion in the promoter domain of the *OsSWEET14* gene^32^. Seven dominant bacterial blight resistance genes including *Xa1*, *Xa3*/*Xa26*, *Xa4*, *Xa10*, *Xa21*, *Xa23*, and *Xa27*, have been cloned and characterized. Among them, *Xa1* encodes an NB-LRR-type protein^36^, *Xa3*/*Xa26* and *Xa21* with LRR receptor kinases (RK)^26,37^, *Xa4* with a cell wall-associated kinase^33^, *Xa10* with a small novel protein harboring 126-amino acid residues and four potential transmembrane helices^28^, *Xa23* with an executor R protein that shares 50% identity to XA10^38^, and *Xa27* with an identical apoplast protein which differ from each other in their promoter regions^39^. It is very interesting that the functional domains of characterized *Xoo* R genes vary so widely. In present study, we anlyzed the candidate resistance genes of *Xa46*(t) between markers RM26981 and RM26984 in the target region based on the information of the MSU Rice Genome Annotation Project Database. Gene prediction results showed that 12 genes were annotated in the linked region. Among them, eight coded for hypothetical proteins, transposon proteins or expressed proteins. No genes encoding transcription factors or proteins similar to known dominant *Xoo* R gene families mentioned above were identified. We identified two genes, Loc_Os11g37540 and Loc_Os11g37550, encoding the serine/threonine-protein kinase (STPK) Doa and calmodulin-2/3/5 as probable candidate genes for *Xa46*(t). Some reports indicated that STPKs encoded genes could participate in plant resistance. Such as the barley STPK gene *Rpg1* provided resistance to stem rust^40^, the powdery mildew resistance gene *Pm21* encoded STPK protein involved in wheat resistance^41^ and the rice STPK *OsPBL1* potentially involved in rice stripe disease resistance^42^ etc.. Several studies have shown that the calmodulin-like genes (CMLs) affect plant immune responses. Expression down-regulation of CML *NtCaM13* in tobacco could enhanc susceptibility to virulent bacteria *Ralstonia solanacearum* and fungi *Rhizoctonia solani* and *Pythium aphanidermatum*^43^, whereas overexpression of pepper CMLs *CaCaM1* could enhance resistance to the pathogens *Xanthomonas campestris pv. Vesicatoria*, *Pseudomonas syringae* and *Hyaloperonospora parasitica*^44^. The tomato gene *APR134* encoding a CaM-related protein is induced in disease resistance when attacked by *Pseudomonas syringae* pv. tomato^45^. Since these reports suggest that STPK and calmodulin-like domain genes might confer resistance against bacterial or fungal pathogens, the two candidates (Loc_Os11g37540 and Loc_Os11g37550) encoding STPK Doa and calmodulin-2/3/5 domain genes were identified as candidates for *Xa46*(t). Surprisingly, only Loc_Os11g37540 was expressed during challenge with bacterial blight, while Loc_Os11g37550 showed no expression. On the other hand, *Xa23*(t) is also mapped on this linked region of Nipponbare reference genome, in which LOC_Os11g37620 is regarded as the target candidate resistance gene of *Xa23*(t)^21^. Further molecular cloning of *Xa23* confirms that it is an executor resistance protein^38^. Nevertheless the expression data showed that LOC_Os11g37620 was not expressed after inoculation in this study.

The region of chromosome 11 where the *Xa46*(t) gene mapped to contains at least the *Xa23* gene was mapped. To clarify if *Xa46*(t) is different from *Xa23*. Sequence comparison of the *Xa23* alleles of H120 and CBB23 suggested that the promoter *EBE*/*ebe* and *CDS* regions of *Xa23* allele of H120 and LTH are different, in which the *EBE*/*ebe* domain of H120 exists 7-bp polymorphism with CBB23, and the *CDS* domain exists one SNP polymorphism between H120 and CBB23. This important domain information defined H120 as haplotype H3 which was susceptible to *Xoo*^23^. So the *Xa46*(t) gene of H120 is not identical to *Xa23*, since H120 is a resistant donor. Characterization of *Xa46*(t) will be helpful to further elucidate the mechanisms of bacterial blight resistance.

Resistance conferred by many bacterial blight and blast R genes can break down when these genes have been widely used for a few years in a large population. Exploitation of more new R genes is urgently needed to solve this problem. The new gene *Xa46*(t) in this study is expected to be very useful in resistance breeding programs since it is resistant against all the pathotypes in Southern China. Many well-known *Xoo* R genes, like *Xa1*, *Xa2*, *Xa10*, and *Xa14* do not confer resistance to *Xoo* pathotypes II, III, IV, and V from Southern China; *Xa3*, *xa8*, *xa13*, and *Xa21* do not confer resistance to pathotypes III, IV, and V; *Xa4* does not confer resistance to pathotype V but does confer resistance to pathotypes I, II, III, and IV. Only *xa5* and *Xa7* confer strong resistance to pathotypes I, II, III, IV, and V^46^. As a recessive *Xoo* resistance gene, *xa5* is difficult to use in hybrid breeding. Moreover, *Xa4* and *Xa7* show specific resistance behaviors related to the receptor background, *Xa7* is not suitable for application in hybrid rice, because the F_1_ generation of its cross with most sterile lines is highly susceptible^47^. In the current study, the resistance conferred by the dominant target gene *Xa46*(t) was independent of genetic background. This gene thus has significant value for improving *Xoo* resistance in hybrid rice, because the F_1_ generations from crosses with susceptible parents, including sterile lines and inbred rice, remain highly resistant (unpublished data). The *Xa46*(t) gene in H120 confers resistance to the six Chinese *Xoo* pathotypes used in this study. We do not know if *Xa46*(t) confers resistance to non-Chinese *Xoo* pathotypes because it is difficult to inoculate plants in segregating populations with many pathogen races. Although it is hard to identify *Xoo* resistant mutants^48^, we were fortunate to screen out simultaneous plural resistance mutations on one M_2_ line derived from LTH. Therefore, it is plausible that *Xa46*(t) confers resistance to all *Xoo* pathotypes.

Interestingly, *Xa* gene-mediated resistance was influenced by genetic background. *Xa21* was reported that its resistance of transgenic lines enhanced than the gene-donor line^37,49^. Different rice cultivars carrying *Xa3*/*Xa26* genes showed variable resistance in different *indica* or *japonica* genetic backgrounds^26^. Among the recessive *Xoo* R genes, *xa33*(t) and *xa42*(t) could be influenced by genetic background^12^. The function of *Xa46*(t) was not influenced by the background of IR24 and CO39, as their F_1_ progeny exhibited dominant resistance to all *Xoo* pathotypes. Thus marker-assisted transfer of the *Xa46*(t) gene to other genetic backgrounds can be expected to enhance the development of the resistant gene carrier under diverse genetic backgrounds.

## Materials and methods

### Plant materials

In our previous study, we had conducted a screening on the bacterial blight resistance of different generations of the mutant lines (T_0_–T_8_) induced by spaceflight, which is derived from a japonica rice cultivar LTH with highly susceptible to bacterial blight disease. A mutant H120 with highly bacterial blight resistance was obtain, and it showed a stable *Xoo* resistance from T_4_ to T_8_, while its wild type LTH showed highly susceptible to *Xoo* (Fig. 1). The IR24 and CO39 cultivars were used as susceptible parents in crosses with H120. The F_1_ progenies from the crosses between H120 and IR24 and CO39 were used to examine whether the *Xoo* resistance gene acted as a dominant or recessive trait. Two F_2_ populations were created from the crosses of IR24/H120 and CO39/H120 for fine mapping of the target *Xoo* resistance gene. The variety LTH, and the International Rice Bacterial Blight (IRBB) near-isogenic lines (NILs) IRBB3, IRBB4, IRBB21, and CBB23 (provided by Chinese Academy of Agricultural Sciences) were used for haplotype analysis. Seeds of these lines were obtained from the International Rice Research Institute, Los Banos, Laguna, Philippines.

### *Xoo* inoculation and evaluation of resistance

Six different virulent strains of *Xoo* from South China were used for evaluation of resistance: pathotype I isolate GD9240, pathotype II GD9269, pathotype III GD9279, pathotype IV GD9315, pathotype V GD9352, and pathotype IX GD9385. The cultivars H120, IR24, CO39, LTH, IRBB1, IRBB2, IRBB3, IRBB4, IRBB5, IRBB, IRBB8, IRBB10, IRBB11, IRBB13, IRBB21, IRBB14, IRBB21, and CBB23 were inoculated with the *Xoo* strains by the leaf-clipping method at the maximum tillering stage under field conditions in Guangzhou, South China^50^. The resistant parent H120, susceptible parents IR24 and CO39, their F_1_ progenies and the F_2_ individuals derived from crosses IR24/H120 and CO39/H120 were inoculated with the pathotype IV isolate GD9315 for genetic analysis. The phenotype was evaluated 20 days after inoculation with the *Xoo* pathogen. The diseased leaf area was evaluated by the Standard Evaluation System for Rice (5th Edition, 2014). In resistance genetic analysis, highly resistant (HR), resistant (R) and moderately resistant (MR) individuals were identified as the resistant phenotype; highly susceptible (HS), susceptible (S) and moderately susceptible (MS) individuals were identified as the susceptible phenotype.

### Molecular mapping analysis using simple-sequence repeat markers

DNA from the parents H120, IR24 and CO39 and the two F_2_ mapping populations of IR24/H120 and CO39/H120 was isolated based on the method of Murray and Thompson^51^. To map the *Xoo* resistance gene(s) from H120, 350 simple-sequence repeat (SSR) markers from the Gramene database (https://www.gramene.org; International Rice Genome Sequencing Project 2005) across the 12 rice chromosomes were screened for parental polymorphism. The polymorphism between the donor parent H120 and recipient parents IR24 and CO39 was analyzed following polymerase chain reaction (PCR) with target region markers (Supplementary Table S5). PCR amplification was performed in 20-μl volumes of reaction mixture containing 30–50 ng template DNA, 10 pmol of each primer, and 10 μl of 2X Super Taq PCRMix (Bioteke). The PCR conditions and detected procedure were referred to Chen et al*.*^52^.

### Markers and target gene linkage analysis

BSA and RCA methods were used to identify polymorphic molecular markers linked to the resistance gene^53,54^. Information of polymorphic markers listed in Supplementary Table S5. Markers and target gene linkage analysis and map construction were conducted with Mapmaker/Exp (version 3.0) with a threshold LOD score of 3.0^55^. The recombination frequencies and map distances were converted into centiMorgans according to the Kosambi function^56^.

### Analysis of putative candidate genes

The genomic sequence between the flanking SSR markers was downloaded from the reference *japonica* rice cv. Nipponbare genome released by the International Rice Genome Sequencing Project and analyzed with the software FGENESH (https://www.softberry.com). All genes with clear open reading frames (ORFs) were analyzed on the basis of the available rice genome sequence and annotation databases from NCBI (www.ncbi.nim.nih.org/unigene) and TIGR release 7.1 (https://rice.plantbiology.msu.edu/). Putative functions for these genes defined in the region of interest were annotated using BLAST-P (https://www.ncbi.nlm.nih.gov).

### Sample preparation and inoculations for RNA sequencing

Cultures of *Xoo* isolate GD9315 were grown at 28 °C on peptone sucrose agar (PSA) with 2 µg/ml tetracycline overnight and diluted in sterile distilled water to 10^8^ cfu/ml. Plant leaves were inoculated with dilutions of both strains and water (for mock) using a shearing inoculation method at the 3–4 leaves stage^52^. Tissue was collected at 0, 6, 12, 24, 48, 72, and 96 h. For bacterial quantification, inoculated leaf tissue was surface sterilized with 10% bleach and rinsed three times with sterile water.

### RNA isolation and quantification

Tissue from all of the samples mentioned above was homogenized using mortar and pestle with liquid nitrogen and RNA was purified using the Plant RNA Purification Kit (NucleoZOL, Gene Company). RNA degradation and contamination were monitored on 1% agarose gels. RNA purity was checked using the NanoPhotometer spectrophotometer (IMPLEN, CA, USA). RNA concentration was measured using Qubit RNA Assay Kit in a Qubit 2.0 Fluorometer (Life Technologies, CA, USA). RNA integrity was assessed using the RNA Nano 6000 Assay Kit of the Bioanalyzer 2100 system (Agilent Technologies, CA, USA).

### Transcriptome sequencing and differential expression analysis

Library preparation and clustering for transcriptome sequencing of all the samples were done by the sequencing company Novogene (China). A total of 1 µg RNA per sample was used as input material for the RNA sample preparations. Sequencing libraries were generated using NEBNext Ultra RNA Library Prep Kit for Illumina (NEB, USA) following manufacturer’s recommendations and index codes were added to attribute sequences to each sample (Novogene Experimental Department). We transferred the original map data to the original sequenced reads with CASAVA base calling through high-throughput sequencing. Phred numerical values were obtained through a probability model in base calling. The clean reads were filtered out from the raw reads for further analysis. The sequencing data quality evaluation is showed in Supplementary Table S1. The index of the reference genome was built using Hisat2 v2.0.4 and paired-end clean reads were aligned to the reference genome using Hisat2 v2.0.4. HTSeq v0.9.1 was used to count the read numbers mapped to each gene. FPKM of each gene was calculated based on the length of the gene and read counts mapped to the gene. Differential expression analysis of two conditions was performed using the DEGSeq R package (1.20.0). The P-values were adjusted using the Benjamini & Hochberg method. Corrected P-values of 0.005 and log_2_ (fold change) of 1 were set as the threshold for significant differential expression. The data analysis of RNA-seq was referred to Conesa et al.^57^. The analysis was divided into three parts: (1) the readcount was first normalized; (2) then the hypothesis test probability (p value) was calculated according to the model; (3) the last multiple hypothesis test correction was made and we retrieved the FDR value.

### Sequence analysis between H120 and CBB23 in the *Xa23* gene domain

To distinguish the relationship between the target gene and the *Xa23* gene, we sequence the H120’s promoter and exon domain of *Xa23* alleles, since the target gene was mapped on the linked *Xa23* region. We download the sequence of *Xa23* gene including the *EBE*_AvrXa23_ (28-bp) and *ORF* (342-bp) sequences in NCBI (National Center for Biotechnology Information, https://www.ncbi.nlm.nlh.gov), then designed several pairs of primers (Supplementary Table S6) to amplify a few sections according to the sequence of *Xa23* with the software FastPCR 6.5.55. Some of the sequencing confirmed primers derived from the paper of Cui et al.^23^. CLUSTAL W was used to analyze sequence alignment (https://myhits.sib.swiss/cgi-bin/clustalw) ^58^. Polymerase Chain Reaction (PCR) was performed in a Gradient iCycler PCR instrument (Bio-Rad) using highly-efficient KOD FX Neo polymerase in a total volume of 25 μl reaction mixture. The reaction mixture contained 50–100 ng of rice genomic DNA (2.0 μl), 10.0 μM of each primer (1.0 μl), 2.0 mM dNTPs (2.0 μl), 2 × PCR KOD buffer (12.5 μl), 0.5 unit (1.0 U/μl) of KOD FX Neo polymerase (0.5 μl), and ddH_2_O (6.0 μl). The PCR profile consists of 3 min initial denaturation at 94 °C, 35 cycles of amplification with 10 s DNA denaturation at 98 °C, 30 s annealing at 60 °C and a final elongation at 68 °C with 1–2 min depending on the length of different fragment. Subsequently, all amplified products were visualized on 1% agarose gels, and the target fragments were purified with DNA Gel Extraction and Purification Kits and sequenced.

### Ethical standards

The experiments comply with the current laws of the country in which they were performed.

## Supplementary information

Supplementary file1
