# Supplementary material for: Identification of the novel bacterial blight resistance gene Xa46(t) by mapping and expression analysis of the rice mutant H120
Source: Sci Rep. 2020 Jul 28;10:12642. doi: 10.1038/s41598-020-69639-y (PMC7387522; doi:10.1038/s41598-020-69639-y)
Supplement: Supplementary file 1 — Supplementary file1 [file 41598_2020_69639_MOESM1_ESM.pdf]

Identification of the novel bacterial blight resistance gene *Xa46*(t) by mapping and expression analysis of the rice mutant H120

Shen Chen, Congying Wang, Jianyuan Yang, Bing Chen, Wenjuan Wang, Jing Su, Aiqing Feng, Liexian Zeng & Xiaoyuan Zhu*

```
                ....|....| ....|....| ....|....| ....|....| ....|....| ....|....|
                    5         15         25         35         45         55
H120            ------TAGC TGCCACGTCA GCTAGGGGAT -GGTAGGCCG GCGCACGGTG GCACAATGTC
CBB23           CTGAGGTAGC TGCCACGTCA GCTAGGGGAT CGGCCCGGCC GC-CACGGTG GCACAATGTC
Consensus             **** ********** **********  **   * *  ** ******* **********

                ....|....| ....|....| ....|....| ....|....| ....|....| ....|....|
                    65        75         85         95         105        115
H120            AGCGCCAGTC CCGTTGGCGC TGACACGGCC AACGTCAGCG CCAATGTATA TGGCGCTGAG
CBB23           AGCGCCAGTC CCGTTGGCGC TGACACGGCC AACGTCAGCG CCAATGTGTT TGGCGCTGAG
Consensus       ********** ********** ********** ********** ******* *  **********

                ....|....| ....|....| ....|....| ....|....| ....|....| ....|....|
                   125        135        145        155        165        175
H120            GCGACGGCCT ATTTTTGGTT GAAGTTTTTG GCAGGGGTTA GTTTCGAAAT AAGTTTTCCA
CBB23           GCGACGGCCT ATTTTTGGTT GAAGTTTTTG GCAGGGGTTA GTTTCGAAAT AAGTTTTCCA
Consensus       ********** ********** ********** ********** ********** **********

                ....|....| ....|....| ....|....| ....|....| ....|....| ....|....|
                   185        195        205        215        225        235
H120            AAAGGGTCAA TTTGTCAAAA AAACGGCTCG TCCCTGAGTC AAAGTCTTCC CTATTAAATT
CBB23           AAAGGGTCAA TTTGTCAAAA AAACGGCTCG TCCCTGAGTC AAAGTCTTCC CTATTAAATT
Consensus       ********** ********** ********** ********** ********** **********

                ....|....| ....|....| ....|....| ....|....| ....|....| ....|....|
                   245        255        265        275        285        295
H120            ATGCGGCATC ACTAACATCA GCTACTATAA AAGTCCCTTC CGCGTCACTA ACATCAGCTA
CBB23           ATGCGGCATC ACTAACATCA GCTACTATAA AAGTCCCTTC CGCGTCACTA ACATCAGCTA
Consensus       ********** ********** ********** ********** ********** **********

                ....|....| ....|....| ....|....| ....|....| ....|....| ....|....|
                   305        315        325        335        345        355
H120            CTATAAAAGT CCCTTCCGCA TCACTAACAT CTTCCTCCCG CATCACTAAC ATCAGCTTCT
CBB23           CTATAAAAGT CCCTTCCG-- ----AAACAT CTTCCTCCCG CATCACTAAC ATCAGCTTCT
Consensus       ********** ********        ***** ********** ********** **********

                ....|....| ....|....| ....|....| ....|....| ....|....| ....|....|
                   365        375        385        395        405        415
H120            ATAAAAGCCC TTCCTTGTTG CATCATCTCA AGGAGCTGCA AGCACTTCCT CTCTGGCAGC
CBB23           ATAAAAGCCC TTCCTTGTTG CATCATCTCA AGGAGCTGCA AGCACTTCCT CTCTGGCAGC
Consensus       ********** ********** ********** ********** ********** **********

                ....|....| ....|....| ....|....| ....|....| ....|....| ....|....|
                   425        435        445        455        465        475
```

```
H120        ACTTCCTCAT CTCAAGGAGT TGCAAATGTT GCATCATCTC AAGGAGCTGG CAGCCGTAGC
CBB23       ACTTCCTCAT CTCAAGGAGT TGCAAATGTT GCATCATCTC AAGGAGCTGG CAGCCGTAGC
Consensus   ********** ********** ********** ********** ********** **********

            ....|....| ....|....| ....|....| ....|....| ....|....| ....|....|
                485        495        505        515        525        535
H120        CGGTATACAC ATGATCCTCA TCTACCTCTG CCGCTTTCTC CTCCGCCGCA GCCGCAACGT
CBB23       CGGTATACAC ATGATCCTCA TCTACCTCTG CCGCTTTCTC CTCCGCCGCA GCCGCAACGT
Consensus   ********** ********** ********** ********** ********** **********

            ....|....| ....|....| ....|....| ....|....| ....|....| ....|....|
                545        555        565        575        585        595
H120        ATTATTCACT GTTTCCAACA GCCTCCGTTT TCGCCTCAAG GTATTAACTG TATTGTTGTA
CBB23       ATTATTCACC GTTTCCAACA GCCTCCGTTT TCGCCTCAAG GTATTAACTG TATTGTTGTA
Consensus   *********  ********** ********** ********** ********** **********

            ....|....| ....|....| ....|....| ....|....| ....|....| ....|....|
                605        615        625        635        645        655
H120        CATATGTCTC TCGGTCATGC TGTTCTACCT GTTTGGCTCC ATCATGCCGC TGCCGCCGTG
CBB23       CATATGTCTC TCGGTCATGC TGTTCTACCT GTTTGGCTCC ATCATGCCGC TGCCGCCGTG
Consensus   ********** ********** ********** ********** ********** **********

            ....|....| ....|....| ....|....| ....|....| ....|....| ....|....|
                665        675        685        695        705        715
H120        GGGCCTCGTG GTCGGTTGGG TCATGGCCCT CATCGCCGTC GAGCTCGCCT ACGCCTTCAT
CBB23       GGGCCTCGTG GTCGGTTGGG TCATGGCCCT CATCGCCGTC GAGCTCGCCT ACGCCTTCAT
Consensus   ********** ********** ********** ********** ********** **********

            ....|....| ....|....| ....|....| ....|....| ....|....| ....|....|
                725        735        745        755        765        775
H120        CTTTCCATAT AGCTTTCGCT ACATCGCTGA CAACGACGAC GACAAGATGG TTATTCTCCC
CBB23       CTTTCCATAT AGCTTTCGCT ACATCGCTGA CAACGACGAC GACAAGATGG TTATTCTCCC
Consensus   ********** ********** ********** ********** ********** **********

            ....|....| ....|....| ....|....| ....|....| ....|....| ....|....|
                785        795        805        815        825        835
H120        TGTTTAAGCC TTCAGGGCCT ATATATATAG TATATATATA AAGCCTTCCA TACTGTCTCT
CBB23       TGTTTAAGCC TTCAGGGCCT ATATATATAG TATATATATA AAGCCTTCCA TACTGTCTCT
Consensus   ********** ********** ********** ********** ********** **********

            ....|....| ....|....| ....|....| ....|....| ....|....| ....|....|
                845        855        865        875        885        895
H120        TCAATAAAGG CTAGCTTGTG TTGTGAGTTG TATCTGTGTA CGTATTTTGT TTGGTTGGTT
CBB23       TCAATAAAGG CTAGCTTGTG TTGTGAGTTG TATCTGTGTA CGTATTTTGT TTGGTTGGTT
```

```
Consensus    ********** ********** ********** ********** ********** **********

             ....|....| ....|....| ....|....| ....|....| ....|....| ....|....|
                  905        915        925        935        945        955
H120         ATATATTGTC ACGTAG-TAT GCCATATATA TATGTATTGC TGTATTTATA TTTGTTACTA
CBB23        ATATATTGTC ACGTAGGTAT GCCATATATA TATGTATTGC TGTATTTATA TTTGTTACTA
Consensus    ********** ****** *** ********** ********** ********** **********

             ....|....| ....|....| ....|....| ....|....| ....|....| ....|....|
                  965        975        985        995       1005       1015
H120         TCTTT-GTTT TTCAGATA-T AAAATTCAGC CAGCCTTGCT -GCTTCGTCG TACGATGTAT
CBB23        TCTTTTGTTT TTCAGATAAT AAAATTCAGC CAGCTTTGCT TGCTTCGTCG TACG-TGTAT
Consensus    ***** **** ******** * ********** **** *****  ********* **** *****

             ....|....| ....|....| ....|....| ....|....| ....|....| ....|....|
                 1025       1035       1045       1055       1065       1075
H120         GCCTCATCAT ATCCTCATCC ATCAGCTGCT CATTAGCTAG CTGGAC-GTG TATATGGTGG
CBB23        GC-TCATCAT ATCCTCATCC ATCAGCTGCT CAT-AGCTAG CTGGGCCGTG T-TATATGTG
Consensus    ** ******* ********** ********** *** ****** **** * *** * ***    *

             ....|....| ....|....| ....|....| ....|....| ....|....| ....|....|
                 1085       1095       1105       1115       1125       1135
H120         TGTTAGTGAA TCAGTCACAT CCATGTAATT CATCCCATGT ATGTTAATTC GTTTTTCATT
CBB23        TGT-AGTGA- TCAGTCACAT CCATGTATTT CATCCCATGT ATGTTAGTTT GTTTTTCATT
Consensus    *** *****  ********** ******* ** ********** ****** **  **********

             ....|....| ....|....| ....|....| ....|....| ....|....| ....|....|
                 1145       1155       1165       1175       1185       1195
H120         TTTGAAAGAA AACATGCATA TATGTTATGA CTCAAAGTTT TGAGATGAAT TTGATCATCT
CBB23        TTTGAAAGAA AACATGCATA TATGTTATAA CTCAAAGTTT TGAGATGAAT TTGATCATCT
Consensus    ********** ********** ******** * ********** ********** **********

             ....|....| ....|....| ....|....| ....|....| ....|....| ....|....|
                 1205       1215       1225       1235       1245       1255
H120         AAGTTGATTG CCATTTGGAA TTATTCTGCT CTGTTGTTGC AACTTTTTTT TTTTTTATGT
CBB23        AATTT-ATTT CCGTTTTGAA TTATTCTGCT CTGTTGTTGC AAACTTTTTT TTT--TATGT
Consensus    ** ** ***  ** *** *** ********** ********** **  ****** ***  *****

             ....|....| ....|....| ....|....| ....|....| ....|....| ....|....|
                 1265       1275       1285       1295       1305       1315
H120         TGGGACACTT TTCCTCCCTC GATTGGATTG GTTATTATTG AAATTTCGTC GATTCATGCT
CBB23        TGGGACACTT TC---TCCTC GGTTTGATTG -TTAT-ATTG AA--TTCGTC GATTCCAGCT
Consensus    ********** *     **** * ** *****  **** **** **  ****** *****  ***
```

```
               ....|....| ....|....| ....|....| ....|....| ....|....| ....|....|
                   1325       1335       1345       1355       1365       1375
H120           AATAATAATA TTTGCCTAGG TTCCACTTCA GAAACAATCA TGC-TAATTG CTGATTCAGG
CBB23          AGTA--TATA TTTGCT--AG TTTCACTC-- -AGACAATCA TGCCTATTTG CTGATCAGGA
Consensus      * **   *** *****    * ** ****     * ******* *** ** *** *****   *

               ....|....| ....|....| ....|....| ....|....| ....|....| ....|....|
                   1385       1395       1405       1415       1425       1435
H120           ACA-TTGAAA ACAACCATTA CAGAGCCAGA CGAAATTAAT TAATTTACAC TCACCAATTA
CBB23          ACAATTGAAA ACAACCATTA CAGAGCCAGA CGAAATTAAT TAATTTACAC TCACCAATTA
Consensus      *** ****** ********** ********** ********** ********** **********

               ....|....| ....|....| ....|....| ....|....| ....|....| ....|....|
                   1445       1455       1465       1475       1485       1495
H120           TTCATCATCA CATCTCTAAA CACCGAATTC TGGGATTCCA TCGTTCGTCC CCTTCCCGCA
CBB23          TTCATCATCA CATCTCTAAA CATCGAATTC TGGGATTCCA TCGTTCGTCC CCTTCCCGCA
Consensus      ********** ********** ** ******* ********** ********** **********

               ....|....| ....|....| ....|....| ....|....| ....|....| ....|....|
                   1505       1515       1525       1535       1545       1555
H120           GCCGTCGGAT CGCGGATCGA CGGTGGCAGA TCGCTTCGTT GAACGTTTTT GTAATATACC
CBB23          GCCGTCGGAT CGCGGATCGA CGGTGGCAGA TCGCTTCGTT GAACGTTTTT GTAATATACC
Consensus      ********** ********** ********** ********** ********** **********

               ....|....| ....|....| ....|....| ....|....| ....|....| ....|....|
                   1565       1575       1585       1595       1605       1615
H120           ATTTCTTGAG GGGGGTTTAT GCAAAATATC TCTATCCCTT ACCTCCTCCT ACCTCGCCGG
CBB23          ATTTCTTGAG GGGGGTTTAT GCAAAATATC TCTATCCCTT ACCTCCTCCT ACCTCGCCGG
Consensus      ********** ********** ********** ********** ********** **********

               ....|....| ....|....| ....|....| ....|....| ....|....| ....|....|
                   1625       1635       1645       1655       1665       1675
H120           CGTGACGCTC TCCTCCCTCA CTCCCATCCG CCGCCGGCGA GGCCCTCTTC GCCGCCGTGT
CBB23          CGTGACGCTC TCCTCCCTCA CTCCCATCCG CCGCCGGCGA GGCCCTCTTC GCCGCCGTGT
Consensus      ********** ********** ********** ********** ********** **********

               ....|....| ....|....| ....|....| ....|....| ....|....| ....|....|
                   1685       1695       1705       1715       1725       1735
H120           CCTCCTCGTC GTCGCTGTAT GGGGCGAG-- ---------- ---------- ----------
CBB23          CCTCCTCGTC GTCGCTGTAT GGGGCGAGGC CGCGGCCCGC GAGGGGCTCG GTGGCGGCGC
Consensus      ********** ********** ********

               ....|....|
                   1745
```

```
H120         ----------
CBB23        GGGGGGATGG
Consensus
```

Supplementary Fig.1 Sequence comparison of *Xa23* alleles between H120 and CBB230

Supplementary Table 1 Protein products of candidate genes.

Genes No.	Protein products
Loc_Os11g37530	Oryza sativa Japonica Group (Japanese rice) hypothetical protein
Loc_Os11g37540	Oryza sativa Japonica Group (Japanese rice) Serine/threonine-protein kinase Doa, putative, expressed
Loc_Os11g37550	Oryza sativa Japonica Group (Japanese rice) Calmodulin-2/3/5, putative
Loc_Os11g37560	Oryza sativa Japonica Group (Japanese rice) expressed protein
Loc_Os11g37570	Oryza sativa Japonica Group (Japanese rice) hypothetical protein
Loc_Os11g37580	Oryza sativa Japonica Group (Japanese rice) hypothetical protein
Loc_Os11g37590	Oryza sativa Japonica Group (Japanese rice) transposon protein, putative, CACTA, En/Spm sub-class
Loc_Os11g37600	Oryza sativa Japonica Group (Japanese rice) transposon protein, putative, CACTA, En/Spm sub-class
Loc_Os11g37610	Oryza sativa Japonica Group (Japanese rice) hypothetical protein
Loc_Os11g37620	Oryza sativa Japonica Group (Japanese rice) hypothetical protein
Loc_Os11g37630	Oryza sativa Japonica Group (Japanese rice) hypothetical protein
Loc_Os11g37640	Oryza sativa Japonica Group (Japanese rice) ADP-ribosylation factor-like protein 5, putative, expressed

Supplementary Table 2 The sequencing data quality evaluation

Sample name	Raw reads	Clean reads	clean bases	Error rate(%)	Q20(%)	Q30(%)	GC content(%)
H120_0	65823390	64916394	9.74G	0.03	97.53	93.37	57.82
H120_6	54913954	54047284	8.11G	0.03	97.53	93.38	56.51
H120_12	55992908	55235918	8.29G	0.03	97.52	93.35	55.42
H120_24	63701086	62621496	9.39G	0.03	97.38	93.04	57.26
H120_48	58803452	57851162	8.68G	0.03	97.56	93.42	57.15
H120_72	61770804	60697432	9.1G	0.03	97.54	93.35	56.8
H120_96	43437362	42672430	6.4G	0.03	97.73	93.8	56.77

Supplementary Table 3 The comparison of RNA-seq reads of H120 and reference genomes of Nipponbare

Sample name	H120_0	H120_6	H120_12	H120_24	H120_48	H120_72	H120_96
Total reads	64916394	54047284	55235918	62621496	57851162	60697432	42672430
Total mapped	61776944 (95.16%)	50938218 (94.25%)	51868621 (93.9%)	59543333 (95.08%)	54996190 (95.06%)	58456988 (96.31%)	40590901 (95.12%)
Multiple mapped	1346915 (2.07%)	1084535 (2.01%)	1198941 (2.17%)	1333339 (2.13%)	1245205 (2.15%)	1337837 (2.2%)	1034384 (2.42%)
Uniquely mapped	60430029 (93.09%)	49853683 (92.24%)	50669680 (91.73%)	58209994 (92.96%)	53750985 (92.91%)	57119151 (94.1%)	39556517 (92.7%)
Read-1	30286633 (46.65%)	24962858 (46.19%)	25384863 (45.96%)	29231634 (46.68%)	26936259 (46.56%)	28611201 (47.14%)	19777340 (46.35%)
Read-2	30143396 (46.43%)	24890825 (46.05%)	25284817 (45.78%)	28978360 (46.28%)	26814726 (46.35%)	28507950 (46.97%)	19779177 (46.35%)
Reads map to '+'	30171257 (46.48%)	24881181 (46.04%)	25263980 (45.74%)	29077562 (46.43%)	26836652 (46.39%)	28543849 (47.03%)	19763331 (46.31%)
Reads map to '-'	30258772 (46.61%)	24972502 (46.2%)	25405700 (45.99%)	29132432 (46.52%)	26914333 (46.52%)	28575302 (47.08%)	19793186 (46.38%)

Supplementary Table 4 The number of genes at different expression levels

FPKM Interval	H120_0	H120_6	H120_12	H120_24	H120_48	H120_72	H120_96
0~1	40817(71.39%)	38438(67.23%)	37685(65.91%)	41229(72.11%)	40575(70.96%)	39862(69.72%)	39770(69.55%)
1~3	4369(7.64%)	3731(6.53%)	3567(6.24%)	4110(7.19%)	4038(7.06%)	3988(6.97%)	3781(6.61%)
3~15	7558(13.22%)	8240(14.41%)	8326(14.56%)	7263(12.70%)	7698(13.46%)	7926(13.86%)	7731(13.52%)
15~60	3266(5.71%)	5118(8.95%)	5699(9.97%)	3257(5.70%)	3531(6.18%)	3957(6.92%)	4406(7.71%)
>60	1168(2.04%)	1651(2.89%)	1901(3.32%)	1319(2.31%)	1336(2.34%)	1445(2.53%)	1490(2.61%)

Supplementary Table 5 Primer sequence of polymorphic microsatellite (SSR) markers used in the study.

Marker name	Forward (5' - 3')	Reverse (5' -3')	PCR[a]	Gel[b]	Products size (bp)	Marker Position
RM26777	AACCTAGGAGCGTTTGTAGATGTGC	GATGCGTTACATCTGGCACTGG	A	I	159	17924318
RM26801	GCCTTCATCCGTAAATCCATAAGC	GAGTACCACATGGCATTATGAGAGC	A	I	166	18578807
RM457	GCACAAGTTGATACTCTCCTCTGACG	CCACCATTATCTGCTCCATCACC	A	I	111	19531155
RM26834	ACGAGTGTATATGGGAACAACC	GCAATCTAGGAGCGTTTATAGG	B	I	260	19266758
RM26988	TCTATTTCTGTCCCTCCTCTTTGG	AGGCCCGACACATAAACAATAGG	A	I	152	22336291
RM206	ATCGATCCGTATGGGTTCTAGC	GTCCATGTAGCCAATCTTATGTGG	B	Ⅱ	477	22480814
RM26820	AACAGATGCACGTCTACACTTTCC	GTGGACTGGAGCTGTGCTAGG	B	I	279	18981546
RM26965	TAAGATGCTAGACCCATCCATGTCG	GACGGAGGGAGTAGCTAGGTAGAGC	B	I	231	21960409
RM26977	GAGCCTCCGCTGAGTCTTCG	CGCCTCCTTCTCCTCTCATCC	B	I	213	22134904
RM26979	ACAACTTCCTCTCTGTTTCACTACCC	CTGCTCTTTGTGCGCTTTATCC	A	I	145	22150068
RM26981	GTCCATGGCTTGGATTTATGAAGG	CATAGAAACAGCTCCATCGTTTGC	B	I	367	22156064
RM26982	ACCAACCCGGACTAAAGATCTCG	TTGGAGGATGAAGTTGCAAACC	B	I	258	22188811
RM26984	ATTCCCACCACAACCAAGATGC	CATGAGGAAGAAGCAGCACAAGC	B	I	258	22221407
RM26985	CACAAGACAACCTTCAATGG	GGCTTAGGAGCGTTTATAGG	A	I	183	22233214
RM26999	CGGCTTCGGCTTCTTCTTCC	GACACTCCCGTTTGTATAACTGTTGC	A	I	173	22640888

[a]A: After preheating 4 min at 94℃, 35 PCR cycles (30 sec at 94℃, 45 sec at 55℃, 1 min at 72℃), followed by 5 min at 72℃. B: After preheating 4 min at 94℃, 35 PCR cycles (30 sec at 94℃, 45 sec at 60℃, 1 min at 72℃), followed by 7 min at 72℃.

[b]I = 8% acrylamide, Ⅱ = 1.2% agrose gel.

Supplementary Table 6 Primers used for amplification and sequencing in this research

Primer name	Primer sequence (5'-3')	Description
A23-F1	TAGCTGCCACGTCAGCTAGG	amplification/sequencing
A23-F2	GCACAATGTCAGCGCCAGTC	amplification/sequencing
A23-F3	AAGTCCCTTCCGCGTCACTA	amplification/sequencing
A23-F4	TCAAGGAGCTGCAAGCACTTCC	amplification/sequencing
A23-R1	CTCGCCCCATACAGCGACGA	amplification/sequencing
A23-R2	GCATACACGTACGACGAAGCAA	amplification/sequencing
A23-R3	GGCCCAGCTAGCTATGAGCA	amplification/sequencing
